# Testing the Independent and Joint Contribution of Exposure to Neurodevelopmental Adversity and Childhood Trauma to Risk of Psychotic Experiences in Adulthood

**DOI:** 10.1093/schbul/sbaa174

**Published:** 2020-12-17

**Authors:** Yiwen Liu, Marina Mendonça, Mary Cannon, Peter B Jones, Glyn Lewis, Andrew Thompson, Stanley Zammit, Dieter Wolke

**Affiliations:** 1 Department of Psychology, University of Warwick, Coventry, UK; 2 Department of Psychiatry, Royal College of Surgeons in Ireland, Dublin, Ireland; 3 Department of Psychiatry, Addenbrooke’s Hospital, University of Cambridge, Cambridge, UK; 4 Institute of Mental Health, University College London, London, UK; 5 Division of Mental Health and Wellbeing, Warwick Medical School, Coventry, UK; 6 Orygen, The Centre of Excellence in Youth Mental Health, Melbourne, Australia; 7 Centre for Academic Mental Health, Bristol Medical School, University of Bristol, Bristol, UK; 8 MRC Centre for Neuropsychiatric Genetics and Genomics, School of Medicine, Cardiff University, Cardiff, UK

**Keywords:** psychosis, bullying, childhood adversity, neurodevelopmental impairment

## Abstract

Exposure to neurodevelopmental adversity and childhood trauma are both independently associated with psychosis. However, there is little research on the mechanism underlying their relationship with each other. The current study investigated both the independent and joint effects of neurodevelopmental adversity and childhood trauma to better understand the etiology of psychosis. A large population-based cohort (*N* = 3514) followed from birth was assessed on psychotic experiences (PE) at 24 years. Neurodevelopmental adversity included obstetric complications (birth weight, gestational age, in-utero influenza exposure, resuscitation) and developmental impairment (cognitive and motor impairments). Trauma exposure included caregiver and peer inflicted trauma up to 17 years. Multiple regression models tested their independent and interactive effect on PE, and path analysis estimated the indirect effect of neurodevelopmental adversity on PE via trauma. Neurodevelopmental adversity (OR = 1.32, 95%CI: 1.08–1.62) and trauma (OR = 1.97, 95%CI: 1.65–2.36) independently increased the odds of PE. There was also an indirect relationship between neurodevelopmental adversity and PE via increased exposure to childhood trauma (β = 0.01, 95%CI: 0.004–0.024). In particular, peer bullying mediated the association between developmental impairment to PE (β = 0.02, 95%CI: 0.01–0.03). In conclusion, children with neurodevelopmental adversity, in particular those with developmental impairment, are more likely to be exposed to trauma. This new etiological understanding of psychosis suggests that PE may be partially modifiable through reducing exposure to peer bullying, especially in children with developmental impairment.

## Introduction

A range of factors have been associated with the development of psychosis, including neurodevelopmental (eg, obstetric complications, premature birth), trauma (eg, bullying), and genetic risks.^1^ Some of these factors have been grouped into meaningful frameworks based on theoretical models to explain the etiology of psychosis. These models include the Neurodevelopmental model (NM) and the Trauma model (TM),^2,3^ 2 of the most widely cited models of psychosis. However, more recently it has been recognized that these models are not mutually exclusive, and the Developmental Risk Factor Model (DRFM) was proposed, which emphasizes the joint effects of neurodevelopmental and trauma-related factors.^4^ It remains unclear what the relative contribution of these factors are and how they may work together in their association with psychosis.

Both neurodevelopmental and trauma-related factors have been implicated in the etiology of psychosis. Brain insults that occur during early development from neurodevelopmental adversity can alter the formation and activity of neural circuits.^5^ Brain abnormalities have been identified in patients with schizophrenia,^6^ and obstetric complications and birth-related factors, such as perinatal infections, premature birth, and low birth weight have all been associated with increased risk of psychosis.^7–9^ Exposure to these early adversities may also lead to cognitive and motor impairments in childhood.^10^ These are often considered as neurological soft signs of psychosis and have been reported to precede the onset of psychosis.^11^

Childhood trauma has also been consistently associated with psychosis.^12^ One putative mechanism is the theory of social defeat, where prolonged exposure to victimization may lead to hostile interpretations of social situations and the intention of others.^13^ Some recent meta-analyses have shown a 2 to 4 times increased risk of psychosis following exposure to any childhood trauma, whether from caregivers (eg, abuse) or peers (eg, bullying)^14,15^ There is also evidence of a cumulative effect, with greater risk associated with increased level/dose of exposure to trauma that persists even when adjusted for genetic risk.^16^

More recently, authors have described how multiple risk factors could impact on a common biological pathway that could lead to psychosis.^4,17^ As proposed by the DRFM, early neurodevelopmental adversity may increase children’s likelihood of being exposed to trauma such as being bullied,^17,18^ which is associated with risk of psychosis. Bullies tend to pick on children with lower cognitive skills and poorer physical health, such as those born prematurely.^19^ However, the model has 2 different interpretations: children with neurodevelopmental adversity may be more vulnerable to the effects of trauma (moderation effect), or they may simply be more frequently exposed to trauma (mediation effect). It could also be a combination of both, and there has been little research examining the mechanism between these risk factors. A previous study found that children at high risk of neurodevelopmental adversity were more frequently exposed to peer bullying, but were not more vulnerable to the effects, providing some evidence that there may be a mediated pathway from neurodevelopmental adversity to psychosis via trauma.^18^

Other risk factors have also been associated with psychosis, including family adversity and genetic risk.^1^ Both common and rare genetic variants have been associated with higher risk of schizophrenia,^20^ and there is some evidence that genetic risk is much greater in people who also had obstetric complications, suggesting that some risk factors act by increasing the likelihood of environmental risks in later life.^21^ Family adversity can also be considered as a risk factor for neurodevelopmental adversity for the child, and maternal psychosocial stress has been associated with neurodevelopmental disorders such as attention deficit hyperactivity disorder.^22^ It is therefore important to account for the role of these risk factors when investigating pathways from neurodevelopmental adversity and trauma to psychosis.

The aim of this prospective longitudinal study from pregnancy to 24 years was to examine the relative contribution of exposure to neurodevelopmental adversity and childhood trauma to the etiology of psychotic experiences (PE). PE are on the psychosis continuum,^23^ and are more prevalent in the population compared to psychotic disorders, which normally requires large sample size to investigate associations. Only one study to our knowledge has investigated this and found a mediated effect of neurodevelopmental adversity on PE via increased exposure to peer bullying.^18^ However, the small sample size in (*N* = 184–399) may have introduced sparse data bias. Preterm birth was also the only proxy for neurodevelopmental adversity; therefore, further research is necessary by using a larger population-based cohort to examine the mechanism underlying the pathway from neurodevelopmental adversity and trauma to PE. First, we examined the direct and independent effects of neurodevelopmental adversity and trauma on PE. Secondly, we investigated their joint effects and the indirect pathway from neurodevelopmental adversity to PE via trauma.

## Methods

### Sample

The ALSPAC cohort is a prospective population study of 14 062 children born to women who resided in the region of Avon, Southwest of UK with expected delivery dates between April 1, 1991 and December 31, 1992. The initial number of pregnancies enrolled is 14 541, combined with enrollment from later phases, the total sample consisted of 13 998 who were alive at 1 year of age. Detailed description of the sample has been reported previously.^24–26^ The study website contains details of all the data that is available through a fully searchable data dictionary and variable search tool (http://www.bristol.ac.uk/alspac/researchers/our-data/). Further phases of recruitment occurred at up to 7, 18, and 24 years; however, only data from the core sample are used in this analysis. In total, 3514 participants completed assessment on PE at 24 years. Ethical approval for the study was obtained from the ALSPAC Ethics and Law Committee and the Local Research Ethics Committees. Informed consent for the use of data collected via questionnaires and clinics was obtained from participants following the recommendations of the ALSPAC Ethics and Law Committee at the time.

### Measures

#### Psychotic Experiences

PE was assessed at 24 years using the semi-structured Psychosis-Like Symptom Interview, which included 12 core questions eliciting key PEs: hallucinations (visual and auditory), delusions (spied on, persecution, thoughts read, reference, control, grandiosity, and other), and thought interference (broadcasting, insertion, and withdrawal).^27,28^ Each question started with a structured stem question asking if the participant had ever had that experience. Participants answering “yes” or “maybe” were cross-questioned to establish whether the experience was psychotic. Coding of PE followed glossary definitions and rating rules for the SCAN (Schedules for Clinical Assessment in Neuropsychiatry). Interviewers rated PEs as not present, suspected, or definitely present. Unclear responses were rated down and only marked as definite when an example met SCAN rating rules. Data was collected and managed using REDCap electronic data capture tools (https://projectredcap.org/resources/citations/). Very good inter-rater and test-retest reliability (kappa = 0.81 and 0.90, respectively) were found similar to previous PE assessments at 12 and 18 years.^27–29^ In this study, PE were dichotomized into none experienced vs any suspected or definite experience present in the past 6 months.

#### Neurodevelopmental Adversity

Neurodevelopmental adversity was derived from a combination of 6 variables, 4 indicating obstetric complications (birth weight, gestational age, influenza exposure during pregnancy, resuscitation at birth), and 2 indicating developmental impairment (IQ and motor impairments). Birth weight was coded as a categorical variable with those either below 2500 grams (low birth weight) or above (normal birth weight). Gestational age was also coded as a categorical variable with those either born below 37 weeks (preterm group) or above (term group). Influenza exposure at any point during pregnancy was obtained from self-reported questionnaires completed by mothers at 18 and 32 weeks of pregnancy and coded as either exposed or not exposed. Resuscitation at birth (via any method) was retrieved from computerized obstetric and neonatal records, coded as either received or not received.

IQ was assessed at age 8 using the short version of the Wechsler Intelligence Scale for Children 3rd UK edition (WISC-III^30^). It was dichotomized so those who scored greater than 1 SD below the mean were classified as having cognitive impairment. Motor skills were also assessed at 8 years using the Movement Assessment Battery for Children (M-ABC^31^), which measured motor performance across 4 different tasks. A final score averaging performances across these tasks was computed for participants who completed all 4 tasks. Those who scored below 15th percentile of the sample were classified as having motor impairment.

Severity of exposure to any neurodevelopmental adversity (any of the 6 variables indicating obstetric complications or developmental impairments) was derived using the following categories: (1) no exposure, (2) 1 exposure, (3) 2 or more exposures. Further sensitivity analysis examined exposure to any indicators for obstetric complications (*N* = 4) separately from developmental impairment (*N* = 2); both were coded as either exposed or not exposed.

#### Trauma

Trauma variables were derived from 121 questions about the frequency and severity of traumatic events up to 17 years of age and has been described previously in detail.^16^ All trauma measures up to 5 years were reported by parents, a mixture of parent and self-report were used from 5 to 11 years, and predominantly child report from 11 to 17 years. Briefly, 5 traumas were considered: physical abuse (physically hurt by caregivers), emotional abuse (saying hurtful things), sexual abuse (any adult or older child forcing or attempting to force participant into sexual activity), emotional neglect (how often caregivers take an interest in aspects of participants’ lives)—all perpetrated by adults, and peer bullying (name-calling, blackmail, assault). Domestic violence was also assessed but not included as the focus is on directly experienced abuse rather than witnessing abuse.

Severity of exposure to trauma was derived using the following categories: (1) no exposure, (2) exposure to 1 trauma, (3) exposure to 2 or more trauma. Specific trauma exposure was also tested in a sensitivity analysis to investigate their independent and joint effect with neurodevelopmental exposure on PE.

#### Confounding Variables

We examined a range of potential confounders. Sex of participants (male or female) was recorded at birth; family history of schizophrenia was assessed via questionnaires sent to both mothers and their partners during pregnancy (or 4 mo post-delivery if mothers were enrolled after 30 wk gestation), and coded as present if either the mother or partner reported a diagnosis. Polygenic risk scores for schizophrenia and bipolar disorder were derived from genome-wide association study (GWAS) using SNPs associated with these outcomes in the discovery sample at p thresholds of 0.05.^32^ Family Adversity Index (short version) during pregnancy consisted of 15 items including maternal age, housing, and financial difficulties, education and marital status, maternal psychopathology, substance use, and crime.^33^ It is a cumulative index developed in the ALSPAC based on Rutter’s indicators of adversity.^34^ Scores ranged from 0 to 15 and were categorized into (1) no adversity (score 0), (2) few adversities (score 1 to 2), and (3) many adversities (score 3 or more). Maternal smoking was assessed via questionnaires during pregnancy and coded as either never smoked or smoked any cigarettes, and maternal age was recorded at delivery.

### Statistical Analysis

#### Primary Analysis

Analyses were conducted using R version 3.6.3. The independent and direct effects of neurodevelopmental adversity and trauma on PE were assessed in multiple logistic regression models, controlling for all confounders and the addition of a multiplicative interaction term. Both variables were coded as an ordered variable with 3 levels (no exposure, 1 exposure, 2 or more exposures) with linear terms. Path analysis was used to examine the indirect effect of neurodevelopmental adversity on PE via increased exposure to trauma, adjusting for the same confounders as above. The “lavaan” package was used in R and the package “semTools” was used to handle missing data using multiple imputation.

### Sensitivity Analysis

A sensitivity analysis was carried out to first investigate obstetric complications separately from developmental impairment; path analysis was used to estimate their indirect effects on PE via any trauma exposure. In a further step, possible indirect pathways from obstetric complications and developmental impairment via each type of trauma (physical abuse, emotional abuse, sexual abuse, emotional neglect, bullying) were also modeled using path analysis. The same packages “lavaan” and “semTools” were used.

### Missing Data

The amount of missing data in predictors and confounders ranged from 0.2% to 26.9%. We used multiple imputations by chained equations with 40 iterations in R, this was used for both multiple logistic regression models (“mice” library) as well as for path analysis (“semTools” library). Data were imputed for all exposure and confounding variables but not for outcome measure of PE (total *N* = 3514). Complete case-analyses are shown in supplementary materials.

## Results

### Sample Characteristics

Descriptive statistics for the sample are shown in table 1. There were more females (62.2%) than males. Around half (54%) had no exposure to neurodevelopmental adversity, 13.5% had 2 or more exposures to neurodevelopmental adversity, and 23.2% were exposed to 2 or more trauma types up to 17 years. The most frequently reported traumas were bullying (29.2%), physical abuse (20.9%), and emotional abuse (20.5%). The prevalence of PE at 24 years was 12.6%.

**Table 1.** Sample Characteristics

		*n*	%
**Exposure to any neurodevelopmental adversity (** ***N*** ** = 3514)**	1 exposure	1143	32.5
	2 or more exposures	475	13.5
***Obstetric complications***	*Exposed*	*1173*	*33.4*
*Birth weight (N = 3470)*	*<2500 g*	*117*	*3.4*
*Gestational age (N = 3514)*	*<37 wk*	*139*	*4.0*
*Influenza exposure during pregnancy (N = 3409)*	*Exposed*	*566*	*16.6*
*Resuscitation (N = 2139)*^a^	*Resuscitated*	*622*	*29.1*
***Developmental impairment***	*Exposed*	*708*	*22.5*
*IQ (N = 2917)*	*<1 SD*	*479*	*16.4*
*Motor skills (N = 2571)*	*<15th percentile*	*315*	*12.3*
**Any trauma exposure (*N* = 3507)**	1 trauma	1067	30.4
	2 or more trauma	814	23.2
*Physical abuse (N = 3504)*	*Exposed*	*731*	*20.9*
*Emotional abuse (N = 3504)*	*Exposed*	*718*	*20.5*
*Sexual abuse (N = 3469)*	*Exposed*	*354*	*10.2*
*Bullying (N = 3427)*	*Exposed*	*999*	*29.2*
*Emotional neglect (N = 3386)*	*Exposed*	*229*	*6.8*
**Psychotic experiences (*N* = 3514)**	Any suspected/definite	443	12.6
**Confounders**			
Sex (*N* = 3514)	Female	2187	62.2
Family Adversity Index (*N* = 3478)	1–2	1355	39.0
	More than 3	271	7.8
Maternal smoking during pregnancy (*N* = 3232)	Yes	388	12.0
Family history of schizophrenia (*N* = 2617)	Yes (either mother or father)	11	0.4
		Mean	SD
Maternal age at birth (*N* = 3514)		29.45	4.58
Standardized genetic risk score (schizophrenia; *N* = 2567)		-0.08	1.01
Standardized genetic risk score (bipolar; *N* = 2631)		0.02	0.99

*Note:* ^a^Resuscitation includes any resuscitation methods used: bag & mask/oxygen, cardiac massage, facial oxygen, intubation, intermittent positive-pressure ventilation with intubation, mouth to mouth & nose, ventilation not otherwise specified, and any other methods.

### Primary Analysis

#### Direct and Moderated Effect

Pooled results from logistic regression model after multiple imputation are shown in table 2. Both exposure to neurodevelopmental adversity and trauma were associated with increased risk of PE in adulthood, even after adjusting for each other and other confounders. Increased exposure to trauma was associated with the largest risk (1.97 odds) of experiencing PE at 24 years. We did not find evidence for an interaction between exposure to neurodevelopmental adversity and trauma in association with PE (table 2).

**Table 2.** Logistic Regression Models Showing the Effects of Neurodevelopmental Adversity and Trauma on PE (*N* = 3514)

	Suspected or Definite PE								
	Unadjusted			Adjusted^a^			Adjusted With Interaction^b^		
	OR	95% CI	*P*-value	OR	95% CI	*P*-value	OR	95% CI	*P*-value
Any neurodevelopmental adversity	1.41	1.16–1.72	***.001***	1.32	1.08–1.62	***.006***	1.32	1.08–1.62	***.008***
Any trauma	2.07	1.74–2.47	***<.001***	1.97	1.65–2.36	***<.001***	2.02	1.66–2.46	***<.001***
Any neurodevelopmental adversity × any trauma	-	-	-	-	-	-	1.11	0.79–1.57	.544

*Note:* ^a^Adjusted for each other as well as confounders: sex, maternal age, maternal smoking, genetic risk score for schizophrenia and bipolar, family history of schizophrenia, and family adversity. Significant confounder: maternal smoking.

^b^Adjusted for each other as well as confounders, with interaction term added.

#### Indirect Effect

**Fig. 1.**
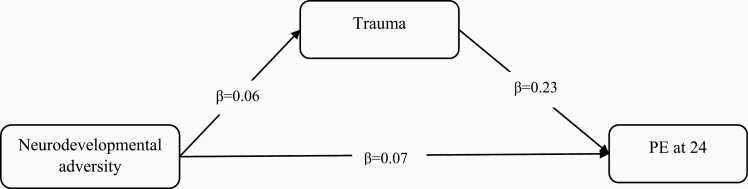
Indirect effect of neurodevelopmental adversity on PE via increased exposure to trauma.

Path analysis found a significant indirect effect of neurodevelopmental adversity on PE via increased exposure to trauma, where trauma mediated the association between neurodevelopmental adversity and PE at 24 years (table 3, figure 1). This indirect effect mediated 17.3% of the total effect of neurodevelopmental adversity on PE.

**Table 3.** Standardized Path Estimates Showing the Direct and Indirect Paths From Neurodevelopmental Adversity to PE Via Trauma (*N* = 3514)

	Standardized Estimates	95% CI	*P*-value
PE at 24 y ~			
Neurodevelopmental adversity	0.065	0.009–0.121	***.022***
Trauma	0.228	0.167–0.290	***<.001***
Trauma (up to 17 y) ~			
Neurodevelopmental adversity	0.061	0.020–0.102	***.003***
Indirect effect			
Neurodevelopmental adversity → Trauma → PE	0.014	0.004–0.024	***.007***
Total effect	0.079	0.023–0.136	***.006***

*Note*: ^a^All paths adjusted for confounders: sex, maternal age, maternal smoking, genetic risk score for schizophrenia and bipolar, family history of schizophrenia, and family adversity. Significant confounders on PE at 24 y: maternal smoking; significant confounders on trauma up to 17 y: maternal smoking, FAI, and genetic risk score for schizophrenia.

### Sensitivity Analysis

**Fig. 2.**
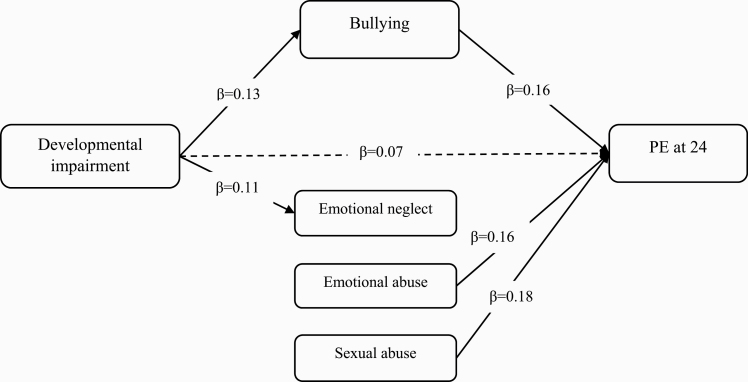
Direct and indirect pathways from developmental impairment and trauma to PE.

When examining specific neurodevelopmental adversity, only developmental impairment, not obstetric complications, predicted PE at 24 years, both directly and indirectly via increased exposure to trauma (supplementary table S1). When specific trauma was examined and adjusted for each other, only peer bullying mediated the relationship between developmental impairment and PE (13.9% of the total effect mediated). Sexual abuse and emotional abuse both had direct effects on PE, but no indirect pathways were found (supplementary table S2, figure 2).

### Complete Case Analyses

Both neurodevelopmental adversity and trauma were associated with increased risk of PE; however, there was weaker evidence for an indirect pathway. When specific trauma was examined, an indirect pathway was found from developmental impairment to PE via peer bullying, consistent with results after imputation (supplementary materials).

## Discussion

The current study examined the independent and joint effects of exposure to neurodevelopmental adversity and childhood trauma to risk of PE in adulthood. Both were independently associated with PE after adjusting for confounders, with trauma having a larger effect on PE than neurodevelopmental adversity. Children exposed to neurodevelopmental adversity were also more likely to experience trauma, which partly mediated the relationship between neurodevelopmental adversity and PE. Under a multiplicative model of risks, those with exposures to both neurodevelopmental adversity and trauma were not more vulnerable to risk of PE. Rather, those with neurodevelopmental adversity experienced trauma more often, which subsequently increased risk of PE in adulthood. Further sensitivity analysis found that it was peer bullying that mediated the relationship between developmental impairment and PE. No indirect pathways were found from obstetric complications or via caregiver-inflicted trauma.

Consistent with previous research, we have shown that exposure to any neurodevelopmental adversity was associated with increased risk of PE in adulthood.^7,8^ These early neurodevelopmental factors may result in disruption to the formation of neural networks, as well as altered inflammatory responses that have been found in people with schizophrenia.^35^ Furthermore, they may manifest in childhood as cognitive and motor impairments, which have been found to precede the onset of psychosis.^11^ However, in a sensitivity analysis, only developmental impairment increased risk of PE when adjusted for all confounders, suggesting that those born with obstetric complications but not developmentally impaired were not at increased risk of PE. Although caution should be used when interpreting the lack of findings for obstetric complications, as maternal influenza exposure was self-reported, while birth weight and gestational age have received mixed findings in the literature with small effect sizes reported.^9^

The association between childhood trauma and PE is also consistent with previous research.^15,16^ Further investigation found that evidence of association with PE was strongest for emotional abuse, sexual abuse, and peer bullying when adjusted for all other trauma types. Emotional and sexual abuse have been frequently reported in the literature as risk factors for psychotic symptoms and disorders.^36^ No association was found for physical abuse after controlling for all other trauma types and confounders; similar findings have been reported previously.^36,37^ Emotional neglect showed no direct effect on PE over and above other indicators of trauma, consistent with the literature that neglect may be a risk factor for general psychopathology rather than psychosis.^36,38^

The association between peer bullying and PE also replicate findings from previous research.^14,18^ Bullying can lead to feelings of social defeat as well as negative evaluations of the self and others, which can increase hostile interpretations of ambiguous events.^13^ Furthermore, only peer bullying mediated the association between developmental impairment and PE. Bullying can be seen as a strategic way of asserting social dominance, and bullies tend to pick on those who are seen as vulnerable such as those with poorer cognitive and motor abilities,^19^ which may explain why those with developmental impairment were more likely to be bullied. This shows the importance of assessing bullying—an area often neglected in assessment of childhood trauma—especially among high-risk groups such as those with developmental impairment.

When testing the mechanism underlying the pathway from neurodevelopmental adversity and childhood trauma to PE, we found an indirect pathway from neurodevelopmental adversity to PE via increased exposure to childhood trauma. Further investigation revealed that the indirect pathway was from developmental impairment via peer rather than caregiver inflicted trauma, suggesting bullies may be more reactive in picking up behavioral impairments resulting from neurodevelopmental adversity. These findings are consistent with a previous study which showed that those born preterm or with low birth weight—as a proxy measure of neurodevelopmental adversity—were more often exposed to peer bullying, which was associated with risk of PE in adulthood.^18^ It suggests a cascade model of psychopathology where early developmental risk may lead to further adversity along the developmental pathway, cumulating in psychopathology.

### Strengths and Limitations

The current study tested the relative contribution of 2 main risk factors of psychosis and included comprehensive measures of neurodevelopmental adversity and childhood trauma to allow for rigorous testing of the relationship between them. The inclusion of a sensitivity analysis also allowed for detailed investigation on the specificity of the association between neurodevelopmental adversity and trauma on PE. Secondly, this study used a large, population-based birth cohort with 24 years of follow-up, with the inclusion of a number of important confounders, including genetic risks. This allowed the demonstration of a temporal relationship between neurodevelopmental adversity, trauma and PE by using prospectively measured variables, consistent with findings from previous prospective studies.^16^

There are also limitations; first, loss to follow-up is inevitable over a 24-year period, and the percentage of missing data in predictor and confounding variables ranged from 0.2% to 26.9%. However, previous research simulating the effects of selective dropout in longitudinal studies found that it may not reduce the validity of predicting outcomes,^39^ and multiple imputation was also used to reduce bias. Results from complete case analysis showed weaker evidence for an indirect effect from neurodevelopmental adversity to PE via any trauma. This could be due to a lack of statistical power as some covariates had large proportion of missing data, leading to a largely reduced sample size in the adjusted analysis. Despite this, an indirect effect was still found from developmental impairment to PE via peer bullying both before and after imputation, offering strong support for the role of peer bullying even in a much smaller sample size. Second, we tested for the moderated and mediated effects of neurodevelopmental adversity and trauma in 2 separate models, thus caution is needed when interpreting the lack of interaction found between these factors. Third, exposure to specific trauma types were coded as binary mediators, and misclassification of the dichotomous mediators may lead to downward bias and underestimation of the indirect effect.^40^ This may lead to some estimation errors, especially in terms of the proportion mediated by the mediator.

## Conclusion

When testing the relative contribution of exposure to neurodevelopmental adversity and childhood trauma to PE, we found that both were independently associated with PE, even after adjusting for socio-demographic and genetic risks. We further found that children exposed to neurodevelopmental adversity were more frequently exposed to trauma, which mediated the relationship between neurodevelopmental adversity and PE. In particular, this mediated pathway was found from developmental impairment and via peer bullying. Regardless of neurodevelopmental adversity, the risk of PE is partially modifiable through reducing childhood trauma. Furthermore, identifying children with developmental impairment may serve as early warning signs for increasing difficulties ahead,^41^ such as being more frequently bullied by peers and subsequently experiencing PE. Interventions should be focused on assessing and reducing bullying in the general population, with a special focus on children at high-risk such as those with developmental impairment.

## Supplementary Material

sbaa174_suppl_Supplementary_Materials
