# Supplementary material for: Testing the Independent and Joint Contribution of Exposure to Neurodevelopmental Adversity and Childhood Trauma to Risk of Psychotic Experiences in Adulthood
Source: Schizophr Bull. 2020 Dec 17;47(3):776–84. doi: 10.1093/schbul/sbaa174 (PMC8084445; doi:10.1093/schbul/sbaa174)
Supplement: sbaa174_suppl_Supplementary_Materials [file sbaa174_suppl_supplementary_materials.docx]

## **Supplementary materials: Sensitivity analysis**

**Table S1.** Standardised path estimates showing the direct and indirect paths from obstetric complications and developmental impairment to PE via trauma (N=3514).

|  | Standardised estimates | 95% CI | p-value |
| --- | --- | --- | --- |
| **PE at 24 years ~** |  |  |  |
| Obstetric complications | 0.046 | -0.011 – 0.103 | 0.112 |
| Developmental impairment | 0.081 | 0.004 – 0.158 | ***0.040*** |
| Trauma | 0.224 | 0.162 – 0.286 | ***<0.001*** |
| **Trauma (up to 17 years) ~** |  |  |  |
| Obstetric complications | 0.022 | -0.019 – 0.064 | 0.286 |
| Developmental impairment | 0.074 | 0.017 – 0.130 | ***0.011*** |
| **Indirect effect** |  |  |  |
| Obstetric complications 🡪 Trauma 🡪 PE | 0.005 | -0.004 – 0.014 | 0.289 |
| Developmental impairment 🡪 Trauma 🡪 PE | 0.016 | 0.003 – 0.030 | ***0.016*** |
| **Total effect** | 0.148 | 0.055 – 0.242 | ***0.002*** |

* All paths adjusted for confounders: sex, maternal age, maternal smoking, genetic risk score for schizophrenia and bipolar, family history of schizophrenia, and family adversity. Significant confounders on PE at 24 years: maternal smoking; significant confounders on trauma up to 17 years: maternal smoking, FAI, and genetic risk score for schizophrenia.

**Table S2.** Standardised path estimates showing the direct and indirect paths from obstetric complications and developmental impairment to PE via specific trauma (N=3514).

|  |  | Standardised estimates | 95% CI | p-value |
| --- | --- | --- | --- | --- |
| **PE at 24 years ~** |  |  |  |  |
| Obstetric complications |  | 0.045 | -0.012 – 0.102 | 0.122 |
| Developmental impairment |  | 0.072 | -0.006 – 0.149 | 0.070 |
| Physical abuse |  | -0.034 | -0.168 – 0.100 | 0.619 |
| Sexual abuse |  | 0.179 | 0.066 – 0.293 | ***0.002*** |
| Emotional abuse |  | 0.163 | 0.052 – 0.273 | ***0.004*** |
| Emotional neglect |  | 0.041 | -0.066 – 0.149 | 0.452 |
| Peer bullying |  | 0.156 | 0.079 – 0.233 | ***<0.001*** |
| **Physical abuse (up to 17 years) ~** |  |  |  |  |
| Obstetric complications |  | 0.038 | -0.013 – 0.089 | 0.140 |
| Developmental impairment |  | 0.029 | -0.042 – 0.100 | 0.424 |
| **Sexual abuse (up to 17 years) ~** |  |  |  |  |
| Obstetric complications |  | 0.035 | -0.028 – 0.098 | 0.275 |
| Developmental impairment |  | 0.037 | -0.050 – 0.124 | 0.402 |
| **Emotional abuse (up to 17 years) ~** |  |  |  |  |
| Obstetric complications |  | 0.014 | -0.038 – 0.066 | 0.593 |
| Developmental impairment |  | -0.015 | -0.087 – 0.057 | 0.689 |
| **Emotional neglect (up to 17 years) ~** |  |  |  |  |
| Obstetric complications |  | -0.072 | -0.144 – 0.001 | 0.052 |
| Developmental impairment |  | 0.106 | 0.014 – 0.199 | ***0.024*** |
| **Peer bullying (up to 17 years) ~** |  |  |  |  |
| Obstetric complications |  | 0.009 | -0.039 – 0.056 | 0.713 |
| Developmental impairment |  | 0.133 | 0.069 – 0.198 | ***<0.001*** |
| **Developmental impairment ~** |  |  |  |  |
| Obstetric complications |  | 0.052 | 0.003 – 0.102 | ***0.038*** |
| **Covariance** |  |  |  |  |
| Physical abuse ~~ Sexual abuse |  | 0.475 | 0.407 – 0.543 | ***<0.001*** |
| Physical abuse ~~ Emotional abuse |  | 0.581 | 0.529 – 0.632 | ***<0.001*** |
| Physical abuse ~~ Emotional neglect |  | 0.076 | -0.018 – 0.171 | 0.112 |
| Physical abuse ~~ Peer bullying |  | 0.145 | 0.08 – 0.210 | ***<0.001*** |
| Sexual abuse ~~ Emotional abuse |  | 0.252 | 0.173 – 0.332 | ***<0.001*** |
| Sexual abuse ~~ Emotional neglect |  | 0.158 | 0.047 – 0.268 | ***0.005*** |
| Sexual abuse ~~ Peer bullying |  | 0.109 | 0.029 – 0.189 | ***0.007*** |
| Emotional abuse ~~ Emotional neglect |  | 0.118 | 0.026 – 0.211 | ***0.012*** |
| Emotional abuse ~~ Peer bullying |  | 0.104 | 0.038 – 0.171 | ***0.002*** |
| Emotional neglect ~~ Peer bullying |  | 0.189 | 0.104 – 0.273 | ***<0.001*** |
| **Indirect effect** |  |  |  |  |
| ***From obstetric complications via ~*** |  |  |  |  |
| Physical abuse |  | -0.001 | -0.007 – 0.004 | 0.645 |
| Sexual abuse |  | 0.006 | -0.005 – 0.018 | 0.293 |
| Emotional abuse |  | 0.002 | -0.006 – 0.011 | 0.591 |
| Emotional neglect |  | -0.003 | -0.011 – 0.005 | 0.463 |
| Peer bullying |  | 0.001 | -0.005 – 0.008 | 0.692 |
| ***From developmental impairment via ~*** |  |  |  |  |
| Physical abuse |  | -0.001 | -0.005 – 0.003 | 0.576 |
| Sexual abuse |  | 0.007 | -0.009 – 0.022 | 0.394 |
| Emotional abuse |  | -0.002 | -0.014 – 0.009 | 0.691 |
| Emotional neglect |  | 0.004 | -0.005 – 0.013 | 0.352 |
| Peer bullying |  | 0.021 | 0.008 – 0.034 | ***0.002*** |
| **Total effect** |  | 0.151 | 0.058 – 0.243 | ***0.001*** |

* All paths adjusted for confounders: sex, maternal age, maternal smoking, genetic risk score for schizophrenia and bipolar, family history of schizophrenia, and family adversity. Significant confounders on PE at 24 years: maternal smoking. Significant confounders for each trauma: physical abuse – family history of schizophrenia, FAI; sexual abuse – sex (female), genetic risk score for schizophrenia; emotional abuse – maternal age, FAI and genetic risk score for schizophrenia; emotional neglect – sex (male), FAI; peer bullying – sex (male), FAI, genetic risk score for bipolar disorder.

**Supplementary materials: Complete case analysis**

## **Table S3:** Logistic regression models showing the effects of neurodevelopmental adversity and trauma on PE (N=1871)

|  | **Suspected or definite PE** | | | | | | | | |
| --- | --- | --- | --- | --- | --- | --- | --- | --- | --- |
|  | **Unadjusted** | | | **Adjusted*** | | | **Adjusted**** | | |
|  | Odds ratio | 95% CI | p-value | Odds ratio | 95% CI | p-value | Odds ratio | 95% CI | p-value |
| **Any neurodevelopmental adversity** | 1.42 | 1.06 – 1.89 | ***0.017*** | 1.37 | 1.02 – 1.83 | ***0.037*** | 1.37 | 1.02 – 1.83 | ***0.037*** |
| **Any trauma** | 2.13 | 1.65 – 2.75 | ***<0.001*** | 2.05 | 1.58 – 2.66 | ***<0.001*** | 2.06 | 1.54 – 2.75 | ***<0.001*** |
| **Any neurodevelopmental adversity x any trauma** | - | - | - | - | - | - | 1.06 | 0.63 – 1.79 | 0.821 |

*Adjusted for each other as well as confounders: sex, maternal age, maternal smoking, genetic risk score for schizophrenia and bipolar, family history of schizophrenia, and family adversity. Significant confounder: maternal smoking.

**Adjusted for each other as well as confounders, with interaction term added

**Table S4.** Standardised path estimates showing the direct and indirect paths from neurodevelopmental adversity to PE via trauma (N=1871).

|  | Standardised estimates | 95% CI | p-value |
| --- | --- | --- | --- |
| **PE at 24 years ~** |  |  |  |
| Neurodevelopmental adversity | 0.112 | 0.009 – 0.214 | ***0.032*** |
| Trauma | 0.238 | 0.158 – 0.318 | ***<0.001*** |
| **Trauma (up to 17 years) ~** |  |  |  |
| Neurodevelopmental adversity | 0.054 | -0.019 – 0.127 | 0.145 |
| **Indirect effect** |  |  |  |
| Neurodevelopmental adversity 🡪 Trauma 🡪 PE | 0.013 | -0.005 – 0.031 | 0.157 |
| **Total effect** | 0.124 | 0.021 – 0.227 | ***0.018*** |

* All paths adjusted for confounders: sex, maternal age, maternal smoking, genetic risk score for schizophrenia and bipolar, family history of schizophrenia, and family adversity. Significant confounders on PE at 24 years: maternal smoking; significant confounders on trauma up to 17 years: maternal smoking, FAI, and genetic risk score for schizophrenia.

**Table S5.** Standardised path estimates showing the direct and indirect paths from obstetric complications and developmental impairment to PE via trauma (N=1784).

|  | Standardised estimates | 95% CI | p-value |
| --- | --- | --- | --- |
| **PE at 24 years ~** |  |  |  |
| Obstetric complications | 0.105 | -0.054 – 0.264 | 0.196 |
| Developmental impairment | 0.115 | 0.010 – 0.219 | ***0.031*** |
| Trauma | 0.231 | 0.150 – 0.313 | ***<0.001*** |
| **Trauma (up to 17 years) ~** |  |  |  |
| Obstetric complications | 0.044 | -0.070 – 0.159 | 0.449 |
| Developmental impairment | 0.034 | -0.043 – 0.110 | 0.393 |
| **Indirect effect** |  |  |  |
| Obstetric complications 🡪 Trauma 🡪 PE | 0.010 | -0.016 – 0.037 | 0.453 |
| Developmental impairment 🡪 Trauma 🡪 PE | 0.008 | -0.010 – 0.026 | 0.393 |
| **Total effect** | 0.238 | 0.052 – 0.423 | ***0.012*** |

* All paths adjusted for confounders: sex, maternal age, maternal smoking, genetic risk score for schizophrenia and bipolar, family history of schizophrenia, and family adversity. Significant confounders on PE at 24 years: maternal smoking; significant confounders on trauma up to 17 years: sex (male), maternal smoking, FAI, and genetic risk score for schizophrenia.

**Table S6.** Standardised path estimates showing the direct and indirect paths from obstetric complications and developmental impairment to PE via specific trauma (N=1773)

|  | Standardised estimates | 95% CI | p-value |
| --- | --- | --- | --- |
| **PE at 24 years ~** |  |  |  |
| Obstetric complications | 0.114 | -0.046 – 0.275 | 0.163 |
| Developmental impairment | 0.099 | -0.006 – 0.203 | 0.065 |
| Physical abuse | -0.075 | -0.263 – 0.113 | 0.435 |
| Sexual abuse | 0.227 | 0.073 – 0.381 | ***0.004*** |
| Emotional abuse | 0.140 | -0.023 – 0.304 | 0.093 |
| Emotional neglect | 0.025 | -0.122 – 0.171 | 0.741 |
| Peer bullying | 0.176 | 0.072 – 0.279 | ***0.001*** |
| **Physical abuse (up to 17 years) ~** |  |  |  |
| Obstetric complications | 0.083 | -0.056 – 0.221 | 0.241 |
| Developmental impairment | -0.002 | -0.095 – 0.091 | 0.968 |
| **Sexual abuse (up to 17 years) ~** |  |  |  |
| Obstetric complications | 0.001 | -0.168 – 0.169 | 0.994 |
| Developmental impairment | 0.033 | -0.080 – 0.147 | 0.568 |
| **Emotional abuse (up to 17 years) ~** |  |  |  |
| Obstetric complications | 0.023 | -0.121 – 0.166 | 0.758 |
| Developmental impairment | -0.439 | -0.141 – 0.054 | 0.383 |
| **Emotional neglect (up to 17 years) ~** |  |  |  |
| Obstetric complications | -0.099 | -0.294 – 0.097 | 0.322 |
| Developmental impairment | 0.079 | -0.045 – 0.203 | 0.213 |
| **Peer bullying (up to 17 years) ~** |  |  |  |
| Obstetric complications | 0.007 | -0.125 – 0.140 | 0.914 |
| Developmental impairment | 0.121 | 0.035 – 0.208 | ***0.006*** |
| **Developmental impairment ~** |  |  |  |
| Obstetric complications | 0.085 | -0.056 – 0.225 | 0.236 |
| **Covariance** |  |  |  |
| Physical abuse ~~ Sexual abuse | 0.476 | 0.388 – 0.564 | ***<0.001*** |
| Physical abuse ~~ Emotional abuse | 0.590 | 0.524 – 0.657 | ***<0.001*** |
| Physical abuse ~~ Emotional neglect | 0.026 | -0.100 – 0.152 | 0.685 |
| Physical abuse ~~ Peer bullying | 0.152 | 0.068 – 0.236 | ***<0.001*** |
| Sexual abuse ~~ Emotional abuse | 0.267 | 0.161 – 0.373 | ***<0.001*** |
| Sexual abuse ~~ Emotional neglect | 0.164 | 0.019 – 0.309 | ***0.027*** |
| Sexual abuse ~~ Peer bullying | 0.140 | 0.037 – 0.242 | ***0.008*** |
| Emotional abuse ~~ Emotional neglect | 0.145 | 0.020 – 0.270 | ***0.023*** |
| Emotional abuse ~~ Peer bullying | 0.115 | 0.027 – 0.203 | ***0.010*** |
| Emotional neglect ~~ Peer bullying | 0.187 | 0.076 – 0.298 | ***0.001*** |
| **Indirect effect** |  |  |  |
| ***From obstetric complications via ~*** |  |  |  |
| Physical abuse | -0.006 | -0.024 – 0.012 | 0.503 |
| Sexual abuse | 0.000 | -0.038 – 0.038 | 0.994 |
| Emotional abuse | 0.003 | -0.017 – 0.024 | 0.762 |
| Emotional neglect | -0.002 | -0.018 – 0.013 | 0.753 |
| Peer bullying | 0.001 | -0.022 – 0.025 | 0.914 |
| ***From developmental impairment via ~*** |  |  |  |
| Physical abuse | 0.000 | -0.007 – 0.007 | 0.968 |
| Sexual abuse | 0.008 | -0.019 – 0.034 | 0.573 |
| Emotional abuse | -0.006 | -0.022 – 0.010 | 0.444 |
| Emotional neglect | 0.002 | -0.010 – 0.014 | 0.758 |
| Peer bullying | 0.021 | 0.002 – 0.040 | ***0.030*** |
| **Total effect** | 0.234 | 0.048 – 0.420 | ***0.014*** |

* All paths adjusted for confounders: sex, maternal age, maternal smoking, genetic risk score for schizophrenia and bipolar, family history of schizophrenia, and family adversity. Significant confounders on PE at 24 years: sex (male), maternal smoking. Significant confounders for each trauma: physical abuse – maternal smoking, FAI; sexual abuse – sex (female); emotional abuse – maternal age (older), FAI and genetic risk score for schizophrenia; emotional neglect – sex (male), family history of schizophrenia, FAI; peer bullying – sex (male), FAI.
